# Structural Mechanism of the Oxygenase JMJD6 Recognition by the Extraterminal (ET) Domain of BRD4

**DOI:** 10.1038/s41598-017-16588-8

**Published:** 2017-11-24

**Authors:** Tsuyoshi Konuma, Di Yu, Chengcheng Zhao, Ying Ju, Rajal Sharma, Chunyan Ren, Qiang Zhang, Ming-Ming Zhou, Lei Zeng

**Affiliations:** 1 Department of Pharmacological Sciences, Icahn School of Medicine at Mount Sinai, New York, NY 10029 USA; 2 Bethune Institute of Epigenetic Medicine, The First Hospital, Jilin University, Changchun, Jilin, 130021 China

## Abstract

Jumonji domain-containing protein 6 (JMJD6) is a member of the Jumonji C family of Fe(II) and 2-oxoglutarate (2OG) dependent oxygenases. It possesses unique bi-functional oxygenase activities, acting as both an arginine demethylase and a lysyl-hydroxylase. JMJD6 has been reported to be over-expressed in oral, breast, lung, and colon cancers and plays important roles in regulation of transcription through interactions with transcription regulator BRD4, histones, U2AF65, Luc7L3, and SRSF11. Here, we report a structural mechanism revealed by NMR of JMJD6 recognition by the extraterminal (ET) domain of BRD4 in that a JMJD6 peptide (Lys84-Asn96) adapts an α-helix when bound to the ET domain. This intermolecular recognition is established through JMJD6 interactions with the conserved hydrophobic core of the ET domain, and reinforced by electrostatic interactions of JMJD6 with residues in the inter-helical α1-α2 loop of the ET domain. Notably, this mode of ligand recognition is different from that of ET domain recognition of NSD3, LANA of herpesvirus, and integrase of MLV, which involves formation of an intermolecular amphipathic two- or three- strand antiparallel β sheet. Furthermore, we demonstrate that the association between the BRD4 ET domain and JMJD6 likely requires a protein conformational change induced by single-stranded RNA binding.

## Introduction

Jumonji domain-containing protein 6 (JMJD6) is a member of the Jumonji C family of Fe (II) and 2-oxoglutarate (2OG) dependent oxygenases^1,2^. The majority of proteins in this family have been assigned as histone lysine demethylases and are involved in chromatin-mediated transcription. The remaining members catalyze protein oxidation and generate a stable hydroxylated modification^3^. JMJD6 has been originally identified as a phosphatidylserine receptor on the cell membrane responsible for phagocytosis of apoptotic cells^4^. However, after JMJD6 was shown through structural bioinformatics to have catalytic activity similar to dioxygenase in the nucleus^5^, JMJD6 was soon described as a bi-functional oxygenase. It is the first discovered arginine demethylase that is able to remove the methyl moieties on methylated arginines of histones (such as H3R2me2 or H4R3me2) and on non-histone proteins, including methylated ERα, RHA, HSP70 and TRAF60^6–8^. JMJD6 also acts as a lysyl hydroxylase by catalyzing C-5-hydroxylation of the splicing regulatory factor U2AF65, of multiple lysine residues of histones H3 and H4, and p53 (on K382), and auto-hydroxylation of internal lysine residues^9^. The initial report on the biochemical role of JMJD6 in histone arginine demethylation had been challenged by other results, which could not verify N-methyl arginine demethylation activity for JMJD6, but rather confirmed JMJD6’s lysine hydroxylation of histone peptide^10^. JMJD6 was also shown to interact with different proteins such as U2AF65, Luc7L3, SRSF11, histones and BRD4^11–14^. Its overexpression is observed in many human malignancies including oral, breast, lung, and colon cancers, suggesting a role in tumorigenesis^15–20^. A biochemical study indicated that JMJD6 can interact with single-stranded RNA (ssRNA)^21^, but not with ssDNA, dsRNA and dsDNA.

Human JMJD6 consists of a JmjC (Jumonji C) domain, three apparent nuclear localization signals (NLS), a DNA binding domain (AT-hook domain), a putative sumoylation site, and a polyserine (polyS) domain^21^. Like the common structural fold of all 2OG oxygenases, JMJD6 contains a distorted double-stranded β-helix (DSBH or cupin) fold that is surrounded by characteristic secondary structure elements. This barrel-type DSBH fold conserves binding motifs for Fe(II) and 2OG oxygenases^21^. However, in comparison to representative structures from other lysine hydroxylase proteins such as JMJD2A and FIH, JMJD6 only contains the similarity of the cupin fold, and is otherwise dramatically different in overall structural conformation from the others, suggesting distinct functions of JMJD6^21^. The JMJD6 structure contains a total of 15 short α-helices with α2, α3, α5, α6, α9, α10, and α11 displaying only one-turn and α4 and α8 two-turns. These one- and two-turn helices are distributed all over the surface of the protein molecule, are loosely connected by a variety of coil loops, and are likely flexible in a solution. These structurally unique small helices of JMJD6 have no clear function, but may be needed to engage interactions with different protein substrates.

Recently, JMJD6 was reported to interact with BRD4^3,7,12^, which is a member of the bromodomains and extra-terminal domain (BET) protein family^22^, and characterized by tandem N-terminal bromodomains (BrDs) followed by an extraterminal (ET) domain^23–25^. BRD4 has important cellular functions in transcription, DNA replication and DNA repair^26,27^. It has also been implicated in development of cancers including acute myeloid leukemia, multiple myeloma, Burkitt’s lymphoma, NUT midline carcinoma, and colon and breast cancers, and is thus recognized as a promising cancer drug target^28,29^. BRD4 and JMJD6 interact with the positive transcription elongation factor b (P-TEFb) complex in its active form to regulate Pol II promoter-proximal pause release for transcriptional activation of a large cohort of genes^7^. BRD4 and JMJD6 interact with each other on distal enhancers, so-called anti-pause enhancers (A-PEs), where JMJD6 is able to demethylate both histone H4R3me2 and the methyl cap of 7SK snRNA. The location of BRD4-JMJD6 on A-PEs binding leads to the dismissal of the 7SK snRNA/Hexim1 inhibitory complex. After removal of repressive histone marks, JMJD6 and BRD4 remain co-bound to anti-pause enhancers and active P-TEFb. Meanwhile, both JMJD6 and BRD4 are capable of attracting and retaining the P-TEFb complex on chromatin, leading to its activation, promoter-proximal Pol II pause release, and transcriptional activation^7^.

Proteomics and knockdown experiments identified that the ET domain of BRD4 is responsible for BRD4 and JMJD6 interaction^12^, but the underlying molecular basis has remained elusive. The ET domain function as a protein interaction domain was first suggested in human BRD2 and BRD4 interactions with the C-terminal segment of Kaposi’s sarcoma-associated herpesvirus (KSHV) latency-associated nuclear antigen (LANA), which facilitates KSHV episome integration into host chromatin for KSHV latency^30^. The ET domain mediates transcriptional activation through recruitment of several cellular transcription regulators including NSD3, ATAD5, CHD4, GLTSCR1, and JMJD6^12,31^. The structures of the BRD4 ET domain bound to MLV Integrase, LANA and NSD3 were recently reported^32,33^, and the general ET domain binding motif and specificity were also explained. However, it has remained unclear how the ET domain selectively interacts with JMJD6 considering its conserved β-sheet cupin core and multiple small unique helices spreading around the protein surface. In this study, we report the NMR 3D structure of the BRD4 ET domain in complex with a peptide derived from one of helices on the JMJD6 surface, and describe detailed structural basis for a unique mode of protein-protein interactions for JMJD6 recognition by the BRD4 ET domain. We further show that BRD4/JMJD6 recognition is likely promoted by ssRNA binding to JMJD6 that induces protein conformational change.

## Results

### JMJD6 Interaction with the BRD4 ET Domain and ssRNA

**Figure 1:**
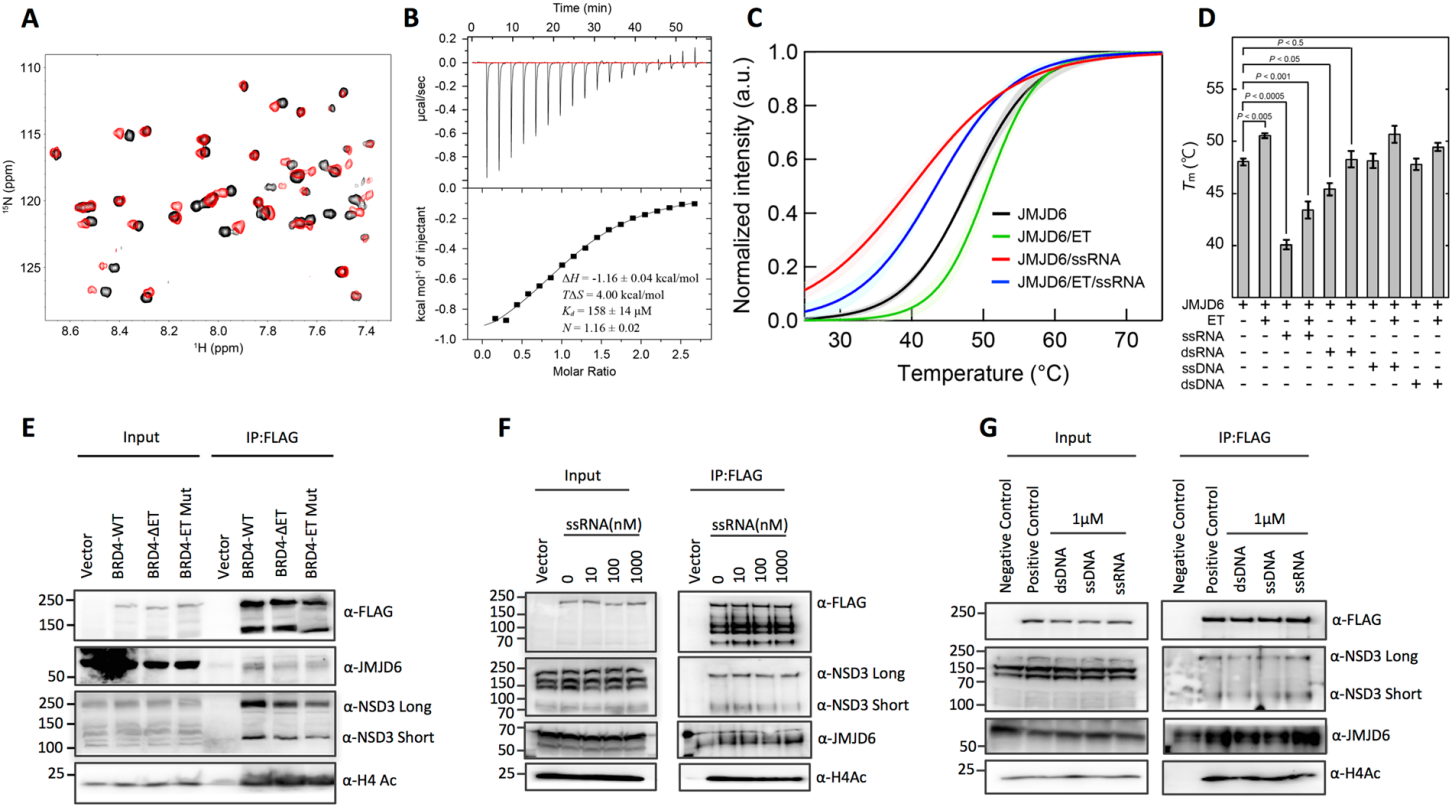
Biochemical analysis of the ET domain of BRD4 binding to JMJD6. (**A)** 2D ^15^N-HSQC spectra of the ET domain in the free form (black) and in complex with the JMJD6 peptide (red). The protein concentration was 0.05 mM and the molar ratio of the protein to peptide was 1:10. **(B)** ITC measurement of the ET domain titrated by the JMJD6 peptide. (**C**) Thermal stability of the full-length JMJD6 determined by differential scanning fluorimetry. The averaged fluorescent intensities were normalized using *T*_m_ and *a* obtained by fitting thermal shift data to a theoretical equation (see Experimental procedures). The profiles in black, green, red and blue were obtained from free JMJD6, JMJD6/ET, JMJD6/ssRNA and JMJD6/ET/ssRNA, respectively. The standard deviations of the profiles were calculated from three independent experiments. In control experiments, samples of only ET, ssRNA, dsRNA, ssDNA or dsDNA without JMJD6 had no fluorescent intensity. (**D**) Thermal stability chart of JMJD6 with/without ET, ssRNA, dsRNA, ssDNA and dsDNA. *T*_m_ is presented as mean ± SEM (n = 3). P-values calculated by the t-test indicate a statistically significant difference. (**E**) HEK293T cells were transiently transfected with Flag-BRD4WT (Flag-BRD4ΔET or Flag-BRD4 ET mutant) and His-JMJD6 (1–403), and cell lysates were harvested 48 h post-transfection. Proteins were separated by SDS-PAGE and immunoblotted with antibodies specific to Flag, NSD3, JMJD6 and H4ac. (**F**) Harvested cell lysates of double-transfected Flag-BRD4WT and His-JMJD6 were added different amounts of ssRNA and followed through the same procedures for Western blotting analysis. At 1 μM ssRNA, the binding of JMJD6 to BRD4 appears much stronger. (**G**) Harvested cell lysates were added 1 μM ssRNA, dsDNA or ssDNA and followed through the same procedures for Western blotting analysis.

The BRD4 ET domain (residues M610-R676) was used in NMR titration experiments to assess its binding to a JMJD6 peptide (residues 84–96, KWTLERLKRKYRN) that resembles the consensus amphipathic ET domain binding motif found in other effector proteins including NDS3 and KSHV LANA^33^. As shown in Fig. 1A, the ^15^N-HSQC spectra of the ET domain displayed backbone amide perturbations induced upon the addition of the JMJD6 peptide. The pattern is nearly identical to that of a longer ET domain construct with the same JMJD6 peptide (data not shown), confirming the conserved core ET domain binding to JMJD6. The affinity of the protein/peptide complex (*K*_*d*_) was determined to be 158 ± 14 μM using isothermal titration calorimetric (ITC) (Fig. 1B), which is similar to that of ET domain/NSD3 complex binding affinity as previously reported^33^. Notably, when the full-length JMJD6 was used in NMR titration, the ^15^N-HSQC spectrum of the ET domain showed severe line broadening, likely induced by conformation exchange, resulting from the ET domain binding to JMJD6 in solution. The solution remains clear, indicating that the protein/protein complex is soluble and likely structured in the solution.

We next conducted a thermal-shift study to characterize the full-length JMJD6 binding to the ET domain. When 100 μM of the ET domain was mixed with 1 μM of the full length JMJD6, the melting temperature of JMJD6 in the ET/JMJD6 complex was increased by 2.5 °C (from 48.0 °C to 50.5 °C) as compared to the free JMJD6 protein, indicating that binding of the ET domain improves the thermal stability of JMJD6 (Fig. 1C,D). Additional thermal-shift experiments with varying ET concentrations (1 μM to 150 μM) and constant JMJD6 concentration show consistent results as well (Supplemental Fig. 1). When we increased the ET domain concentration above 150 μM, we observed small shifts of fluorescence intensity due to possible ET domain binding to the SYPRO dye. Notably, we added ssRNA (1 μM) into JMJD6. The binding to ssRNA contributed JMJD6 to a 7.9 °C decrease in melting temperature as compared to free JMJD6 (from 48.0 °C to 40.1 °C) (Fig. 1C). When we mixed ET domain into the JMJD6/ssRNA complex, JMJD6 melting temperature resulted in an increase of +3.3 °C (from 40.1 °C to 43.4 °C), which was slightly better than the free JMJD6 binding with ET. These results indicate that binding of ssRNA likely makes JMJD6 structurally more flexible, and the structural stability of JMJD6/ssRNA can still be improved upon the ET domain binding. The increased conformational flexibility of the JMJD6/ssRNA complex explains the difficulty in obtaining crystals of the complex for detailed structural analysis in the previous study^21^. Notably, as from our thermal shift binding analysis in Fig. 1D, we observed that JMJD6 weakly interacts with dsRNA with or without the ET domain, but does not bind to either ssDNA or dsDNA.

### JMJD6 Interaction with BRD4 in Cells

To validate the binding activity between JMJD6 and the BRD4 ET domain, we generated a domain-specific deletion that removes the ET domain (BRD4ΔET) in the context of full-length BRD4 and we utilized stable HEK293T cells expressing Flag-tagged wild type BRD4 (BRD4WT), BRD4ΔET and BRD4 ET mutant (E651A/E653A) as well as His-tagged JMJD6 (1–403) to perform immunoprecipitations (IP) followed by Western blotting with antibodies for Flag-tag, JMJD6, NSD3 and H4ac. As shown in Fig. 1E, we confirmed the binding of transfected BRD4WT to JMJD6, NSD3 and H4ac, whereas transfected BRD4ΔET and ET mutant showed markedly weaker association to JMJD6 and NSD3 than the wild type BRD4, but had the same level of binding to H4ac. These data confirm the importance of BRD4 ET domain for mediating the interaction with JMJD6 or NSD3 proteins. In addition, we examined BRD4 and JMJD6 interactions in HEK293T cells with endogenous proteins, or with single- or double-transfected Flag-BRD4WT, Flag-BRD4ΔET, Flag-BRD4 ET mutant and His-JMJD6. While endogenous BRD4 and JMJD6 interactions were very weak, we were able to clearly detect their interactions in the double-transfected IP experiment (Supplemental Fig. 1C).

To determine whether ssRNA can enhance JMJD6 and ET domain interaction, we choose the same 27-nt ssRNA probe used in a previous JMJD6 study^21^. HEK293T cells were double-transfected with Flag-BRD4 and His-JMJD6, and different amounts of ssRNA and RNase inhibitors were added to cell lysates, followed by the same IP protocol and Western blotting analysis. As shown in Fig. 1F, the association of Flag-BRD4WT with JMJD6 is much stronger when ssRNA concentration is at 1 μM than the positive control, but the binding to NSD3 appears to be weaker when ssRNA concentration increases, while binding to H4ac remains unchanged. In addition, we added the same amount of dsDNA or ssDNA (36-bp or 36-nt) to the cell lysate for comparison, but the results showed no improvements on JMJD6 interaction with BRD4. The endogenous and single-transfected IP experiments also have no additional effect with the addition of ssRNA, dsDNA and ssDNA. These data demonstrate that ssRNA binds to JMJD6 and contributes positively for JMJD6 binding to BRD4, which confirm the similar observations in our thermal shift experiment.

### Structure of the BRD4 ET Domain/JMJD6 peptide Complex

**Figure 2:**
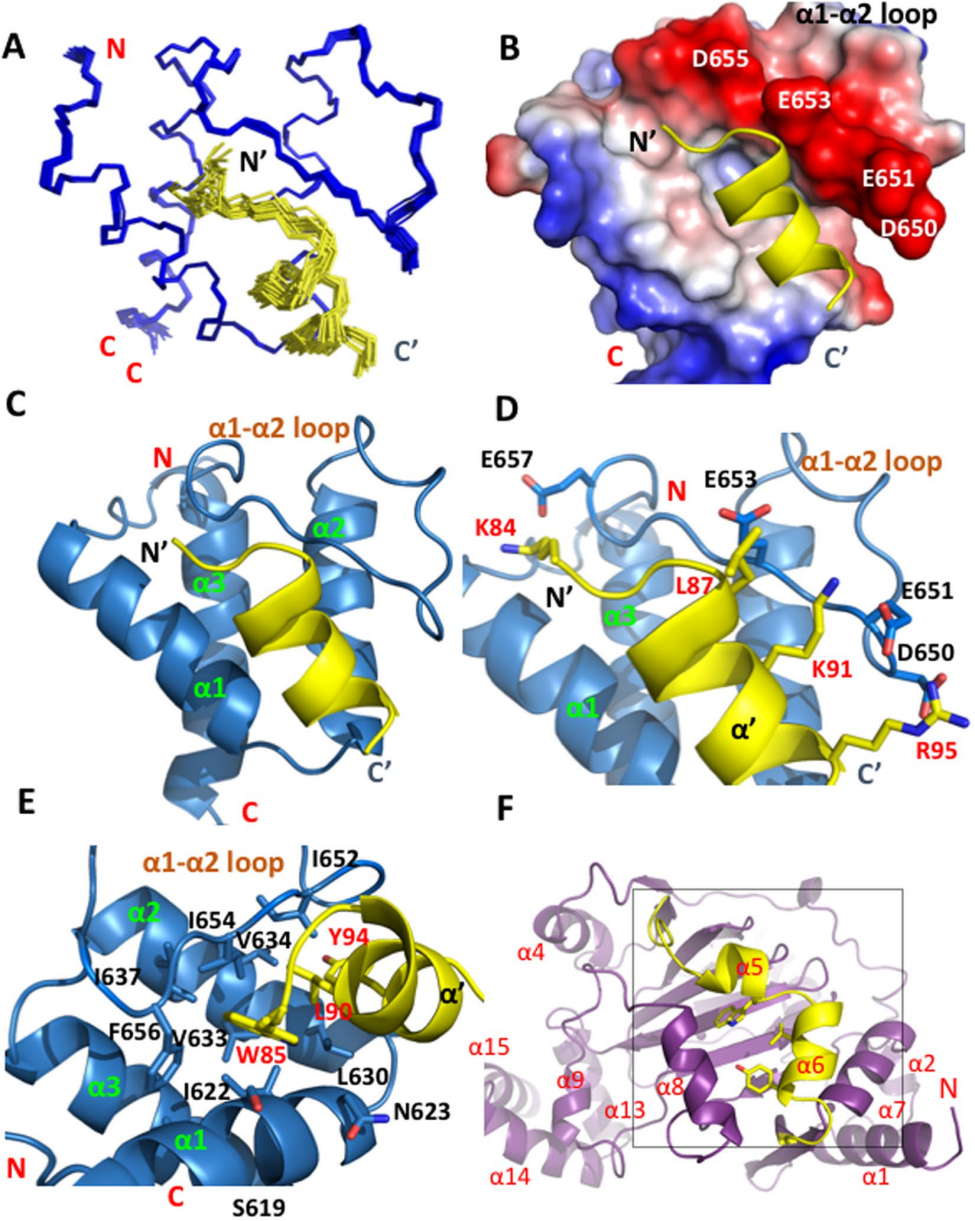
Structure of the BRD4 ET domain with JMJD6 complex. **(A)** The backbone atom superposition of the final 20 NMR-derived structures of the complex. The figure shows the BRD4 ET domain residues 601–683 (blue) and the JMJD6 peptide residues 84–96 (yellow). The terminal residues, which are structurally disordered, are omitted for clarity. (**B**) Electrostatic potential surface representation of the BRD4 ET domain bound to the JMJD6 peptide (yellow). The orientation is the same as (**A**). The electrostatic potential calculation was performed in PyMol (v1.8) using the APBS modules. (**C**) Ribbon depiction of the lowest energy NMR structure of the BRD4 ET domain/JMJD6 peptide complex with the same orientation of (**A**). (**D**) The stick diagram showing side-chain interactions of negative charged residues in the BRD4 ET domain α1-α2 loop (D650, E651, E653 and E657) with positive charged residues of JMJD6 peptide (K84, K91 and R95). ET domain residues involved in peptide binding are labeled and colored in blue, and JMJD6 residues in yellow, the orientation is the same as (**A**). **(E)** Expanded diagram of key hydrophobic side-chain interactions from the α-helix JMJD6 peptide to the hydrophobic binding core of the BRD4 ET domain surrounded by α1, α2 helices and α1-α2 loop. The peptide in the stick representation is depicted as in the ribbon diagram on (**D**). The peptide elements are colored in yellow and red. The protein hydrophobic side-chains are colored in blue. **(F)** Ribbon depiction of the crystal structure of the JMJD6 with the orientation of the α6 helix facing front (colored in yellow).

We determined the three-dimensional (3D) structure of the BRD4 ET domain (residues 601–683) in complex with a JMJD6 peptide (residues 84–96, KWTLERLKRKYRN) by using triple-resonance NMR spectroscopy methods^34^. The solution structure of the protein/peptide complex was determined using a total of 2199 NMR-derived distance and dihedral angle restraints (Table 1). Superposition of an ensemble of the 20 final structures of the complex is depicted in Fig. 2A, and the final RMSDs are 0.18 ± 0.03Å and 0.48 ± 0.07Å for secondary backbone and heavy atoms, respectively (Table 1). The protein structure consists of the three-α-helix bundle with a long loop connecting α1 and α2 (α1-α2 loop) extended across the surface of the entire protein (Fig. 2B,C). The structure also reveals that the JMJD6 peptide retains an α-helical conformation similar to that (α6) in the crystal structure of the full-length JMJD6 protein. Superposition of the free BRD4 ET domain structure (PDB: 2JNS) and JMJD6 peptide bound complex yields an RMSD of 0.81Å, indicating that the ET domain fold is maintained upon JMJD6 peptide binding.

**Table 1:** Summary of restraints and statistics of the final 20 out of 200 structures of the BRD4 ET domain in complex with JMJD6 peptide.

	JMJD6
Protein NMR distance and dihedral constraints	
Distance constraints	
Total NOE	2199
Intra-residue	732
Inter-residue	1467
Sequential (|i−j| = 1)	435
Medium-range (1 < |i−j| ≤ 5)	573
Long-range (|i−j| > 5)	459
Inter-molecular constraints	143
Hydrogen bonds	43
Total dihedral angle restraints	
Phi angle	85
Psi angle	85
Ramachandran Map Analysis (%)^a^	
Most favored regions	100.0
Additional allowed regions	0.0
Generally allowed regions	0.0
Disallowed regions	0.0
Structure statistics	
Violations (mean +/− s.d.)	
Distance constraints (Å)	0.047 +/− 0.0014
Dihedral angle constraints (°)	0.36 +/− 0.060
Max. dihedral angle violation (°)	0.48
Max. distance constraint violation (Å)	0.050
Deviations from idealized geometry	
Bond lengths (Å)	0.0051 +/− 0.00010
Bond angles (°)	0.61 +/− 0.013
Impropers (°)	1.5 +/− 0.068
Average pairwise r.m.s. Deviation (Å)^b^	
Heavy	0.48 +/− 0.066
Backbone	0.18 +/− 0.034

^a^Procheck calculation was done for protein residues 608–640, 653–676.

^b^The residue number ranges used in full molecule pairwise root-mean-square (r.m.s.) deviation calculations consists of 609–676.

^c^Pairwise r.m.s. deviation was calculated among top 20/200 lowest energy structures.

The JMJD6 peptide/ET domain recognition interface covers approximately 1600 Å^2^ of solvent-accessible surface area of the protein, which consists of negatively charged exterior residues Asp650, Glu651, Glu653, Asp655, and Glu657 in the α1-α2 loop, and the interior hydrophobic core residues from α1 and α2 in their inter-connecting α1-α2 loop (Fig. 2B,C). These residues engage in extensive hydrophobic and electrostatic interactions with the two-turn amphipathic α-helix of the JMJD6 peptide. Specifically, side chains of Asp650, Glu651, Glu653, and Glu657 of the ET domain form electrostatic interactions with side chains of Arg95, Lys91 and Lys84 of JMJD6 (Fig. 2D). Moreover, three hydrophobic and aromatic residues of JMJD6, Trp85, Leu90, and Tyr94, are intercalated into the hydrophobic core of the protein, interacting with Ile652, Ile654 and Phe656 in the α1-α2 loop, and also with Ile622, Leu630, Val633, Val634 and Ile637 from α1 and α2. In particular, we observe a large number of intermolecular NOEs for the aromatic ring of Trp85 of JMJD6 with Ser619, Ile622, Val633, Ile637, Ile654 and Phe656 of ET; Leu90 of JMJD6 with Ile622, Asn623, Leu630, Val633, Ile652 and Ile654; and Tyr94 of JMJD6 with Ile622, Leu630, Val634 and Ile652, respectively (Fig. 2D,E). Additionally, side-chain methyl groups of Leu87 of JMJD6 exhibit NOEs to Glu653 of the protein. Unlike ET domain recognition of NSD3 or other effector proteins that is accomplished through the formation of an intermolecular antiparallel β-sheet^32^, there are no observed hydrogen bonds between the backbone atoms of the JMJD6 peptide and the ET domain protein, as they would be manifested by slow exchange amide protons in H-D exchange experiments.

To understand the relative contributions from these key residues of the JMJD6 peptide in interactions with the BRD4 ET domain, we designed five mutant JMJD6 peptides and tested them in ITC and 2D ^15^N-HSQC experiments. ITC with the mutant peptides show that they are weaker in binding to the ET domain than the wild type peptide (Supplementary Fig. 2). The K_d_ of the W85A peptide is 675 ± 66 μM, about four-fold weaker than that of wild type peptide, while the L90A, K91A and R95A mutants nearly abolish the binding to the ET domain. These results are confirmed by ^15^N-HSQC titration studies (Supplemental Fig. 3). Further, the K91A and R95A mutants also show markedly reduced binding by ITC as well as by ^15^N-HSQC. The latter method is more sensitive in measuring weak protein-peptide binding than ITC. The Y94A mutant peptide was not soluble in solution and could not be used in ITC and ^15^N-HSQC experiments. Collectively, our data demonstrate that key residues Trp85, Leu90, Tyr94, Lys91 and Arg95 of JMJD6 are crucial to BRD4 ET domain binding. Notably, the three key residues (Trp85, Leu90 and Tyr94) on the α6 helix in the JMJD6 crystal structure are not completely exposed to exterior solvent, they instead partially interact with the α8 helix (Fig. 2F). However, in the solution state, the α6 helix can be more flexible due to its loose connections through coil loops. Furthermore, the conformation of JMJD6 can be further altered upon ssRNA binding to the back-side of the protein as indicated by melting temperature decreases in thermal-shift experiments and improving binding of JMJD6 to BRD4 in IP experiments.

**Figure 3:**
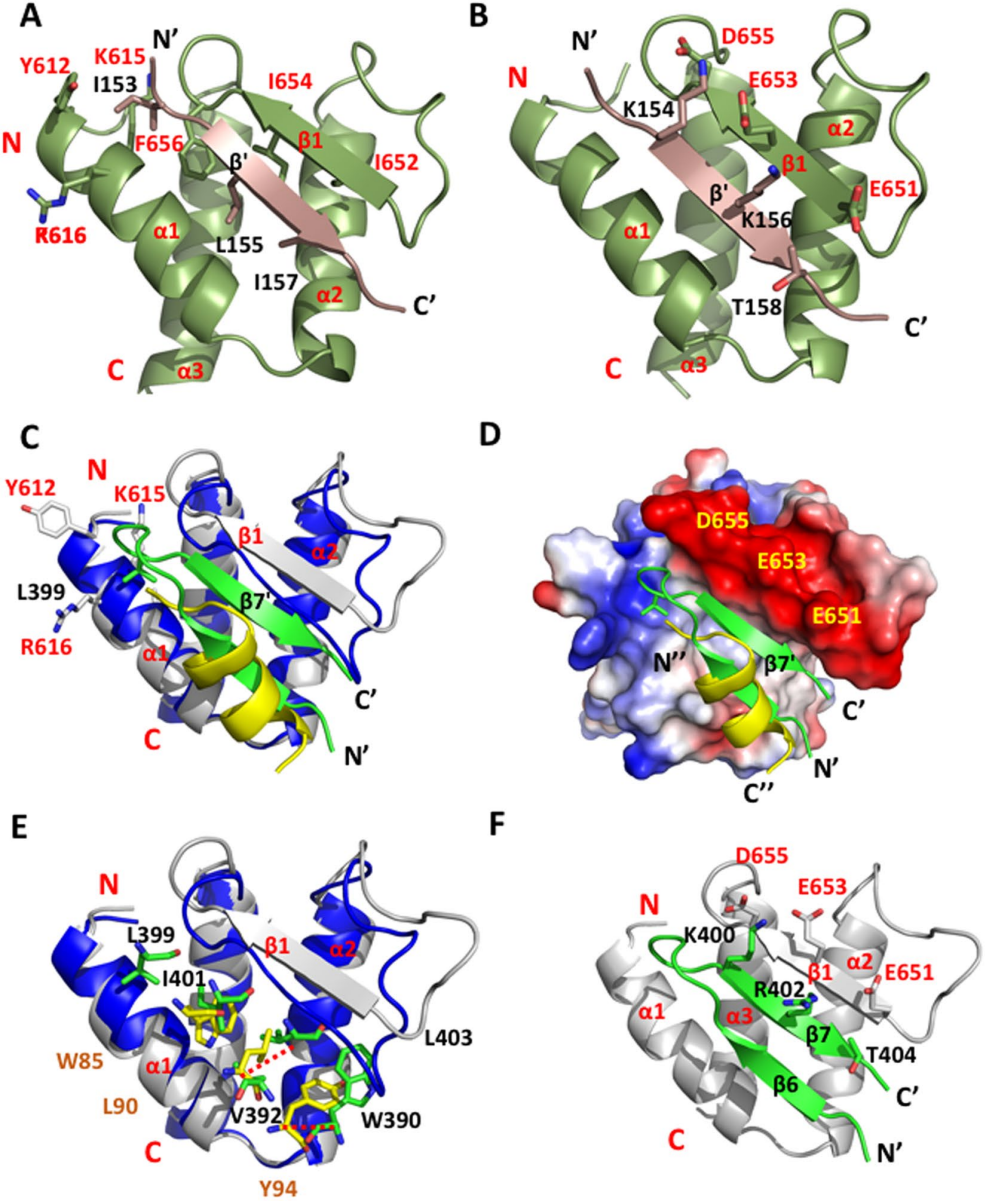
Structural comparison of the BRD4 ET domain bound to NSD3, MLV-IN and JMJD6 peptides. **(A)** BRD4 ET domain/NSD3 complex. Key hydrophobic side-chain interactions from the NSD3 with the hydrophobic core of the ET domain. The peptide elements are colored in brown, and the protein hydrophobic side-chains are colored in green. **(B)** Negative charged residues on β1-strand of ET electrostatically interact with NSD3 peptide. **(C)** Superimposed ribbon diagram of BRD4 ET/MLV-IN and BRD4 ET/JMJD6 complexes. The β1-strand of ET in MLV-IN complex undergoes expansion to open up the binding pocket for the three-strand β sheet conformation with the MLV-IN peptide. **(D)** Electrostatic potential surface representation of the BRD4 ET/MLV-IN (green), and the superimposed JMJD6 relative position compared to MLV-IN peptide. The orientation is the same as (**C**). The electrostatic potential calculation was performed in PyMol (v1.8) using the APBS modules. **(E)** Key hydrophobic residues of JMJD6 in comparison to relative positions of the corresponding residues of MLV-IN, and MLV-IN residues are about 3.9 Å deeper into the pocket and involve more extended interactions. The red dash lines show the distances between the corresponding residues. **(F)** Side-chain interactions of negative charged residues in the β1-strand of ET domain with positive charged residues of MLV-IN peptide. The residues involved and electrostatic effects are similar to the NSD3 binding in (**B**). ET domain residues involved in peptide binding are labeled and colored in grey, and MLV-IN residues in green.

### Comparison to the BRD4 ET domain/NSD3 complex

Our new structural analysis indicates that the conserved three-helical bundle fold of the ET domain is functionally versatile, capable of engaging effector protein recognition either through an antiparallel β-sheet formation (for NSD3, LANA, and MLV-IN-EBM), or through helix to helical bundle interactions (for JMJD6) as shown in this study. As compared to the structure of the BRD4 ET domain/JMJD6 complex, the structure of the ET domain/NSD3 complex (residues 152–163, EIKLKITKTIQN; PDB ID 2NCZ)^32^ shows that the NSD3 peptide forms a two-stranded anti-parallel β sheet with the protein residues in the region connecting α1 and α2 (Fig. 3A). Only two hydrophobic residues Leu155 and Ile157 of NSD3 insert into the hydrophobic core of the protein interacting with Ile654 and Ile652 of the β1 strand and the rest of the hydrophobic residues of Ile622, Asn623, Leu630, Val633 and Val634 on α1 and α2 helices (Fig. 3A); two positively charged Lys154 and Lys156 of NSD3 form electrostatic interactions with corresponding negative Asp655 and Glu653 (Fig. 3B). In addition, Thr158 of NSD3 forms hydrophobic interactions with Glu651, and Ile153 is bound in a small hydrophobic grove surrounded by Tyr612, Lys615, Lys616 and Phe656 of ET domain (Fig. 3A,B). Three hydrogen bonds are observed between the two intermolecular anti-parallel β strands.

Notably, although ITC results show similar affinity of BRD4 ET binding to JMJD6 and NSD3 peptide (JMJD6, *K*_*d*_ of 158 ± 14 μM; and NSD3, *K*_*d*_ of 140.5 ± 2.2 μM), the underlying thermodynamic mechanisms are apparently different. As revealed by the thermodynamic parameters determined by ITC, the free energy change (ΔG = 5.16 kcal/mol) of the ET domain binding to the JMJD6 peptide is contributed collectively by a favorable entropy change (TΔS = 4.00 kcal/mol) and a small favorable change in enthalpy (ΔH = −1.16 kcal/mol), whereas the ET domain binding to the NSD3 peptide (ΔG = 5.15 kcal/mol) is driven by a dominated favorable entropy change (TΔS = 8.0 kcal/mol) that overcomes a moderately unfavorable enthalpy change (ΔH = 2.85 kcal/mol). The binding of BRD4 ET with the LANA peptide is almost identical, structurally and thermodynamically, to the NSD3 peptide^32^, which is consistent with their mode of ligand recognition.

### Comparison to the BRD4 ET domain/MLV-IN-EBM complex

The BRD4 ET domain recognition of a MLV-IN-EBM (MLV IN) peptide (residues 389–405, PDB ID: 2N3K) is thermodynamically similar to the ET domain association with JMJD6^32^, but the affinity of the former (*K*_*d*_ of 159 ± 12 nM, determined by ITC) is stronger than the latter (1000x). The MLV IN peptide recognition by the ET domain features more extensive contact by a three-stranded intermolecular anti-parallel β-sheet. As compared to the ET/JMJD6 complex, the β1 strand of the ET domain when bound to the MLV IN peptide is pushed outward to provide greater interface for intermolecular interactions (Fig. 3C,D). As shown in superposed structures, the α-helix of JMJD6 peptide is in the same position as the β6′ of the MLV peptide, while the α1-α2 loop of the ET domain in the JMJD6-bound form is not as open as the β1 strand in the MLV-bound form, indicating that MLV binding occupies greater interface area than JMJD6, which requires the protein to undergo larger conformational changes. Accordingly, three aromatic and hydrophobic residues Ile401, Leu403 and Trp390 of MLV interact by an average of 3.9 Å deeper than the corresponding JMJD6 residues Trp85, Leu90 and Tyr94 with the hydrophobic core of the protein (Fig. 3E); residue Val392 of MLV lies directly on top of α1 helix of the protein; Leu399 of MLV is in the same small grove outside the main pocket as that of Ile153 from NSD3 peptide (Fig. 3C,E). In addition, positively charged residues Lys400 and Arg402 as well as Thr404 participate in electrostatic interactions with exterior residues of D655, E653 and E651 of the protein, which are characteristic of the β-sheet conformation and similar to those of corresponding residues Lys154, Lys156 and Thr158 of the NSD3 peptide (Fig. 3F). These results explain the higher affinity of MLV IN peptide binding to the ET domain in comparison to the JMJD6 peptide.

## Discussion

JMJD6, a jumonji C domain-containing protein demethylase and hydroxylase, has been associated with multiple biological processes, including regulation of transcription and splicing via post-translational modification^6,10,11^. However, the molecular mechanisms by which it engages in these processes have remained elusive. BRD4 plays important roles in multiple cellular functions, such as transcriptional elongation, epigenetic regulation, and DNA repair^7^. Its functional importance in the development of multiple diseases has supported its recognition as an attractive drug target. Previous work has shown that the interaction of BRD4 and JMJD6 with the positive transcription elongation factor b (P-TEFb) complex regulates Pol II promoter-proximal pause release, leading to activation of a large cohort of genes^7^. The interaction of BRD4 with JMJD6 was found to occur through the BRD4 ET domain^7,12^, but the structural details of this interaction were not clearly understood.

In our study, we provide the structural mechanism of JMJD6 recognition by the ET domain of BRD4, which is distinctly different from that of the ET domain recognition of other effector proteins including NSD3, LANA of herpesvirus, and integrase of murine leukemia virus, which feature the formation of an intermolecular antiparallel β sheet^32,33^. This demonstrates the functional versatility of the conserved three-helix bundle fold of the ET domain for modulating protein-protein interactions, which likely facilitate its ability to engage in multivalent interactions involving not only protein-protein but also protein-RNA interactions. Our results suggest that the ET domain recognition of the α6 helix of JMJD6 requires significant structural re-arrangement of the latter, which is likely made possible through allosteric effects of JMJD6 binding to single-stranded RNA. Surprisingly, this effect was not observed with DNA. We speculate that active transcription of these enhancers, leading to generation of nascent ssRNA, may need to occur prior to JMJD6 and BRD4 interaction and/or recruitment. Understanding the dynamics of this interaction, as well as the role that this plays in regulation of transcription by enhancers, will require a combination of both structural analysis and functional epigenetics. Our study presented here provides a clear guidance for further mechanistic characterization of the multifaceted functions of JMJD6 in coordination with BRD4 and other proteins in regulation of transcription during various normal and pathologic processes in biology.

## Experimental Procedures

### Sample Preparation

The human BRD4 ET domain (Residues 601–683, or 610–676) and full-length JMJD6 subcloned in pNIC28 vector were expressed and purified using a procedure as described previously^21,33^. Briefly, His-Tagged BRD4 ET domain and His-Tagged JMJD6 were overexpressed in Escherichia coli pRIL plasmid BL21-CodonPlus cells and induced with 0.3 mM isopropyl-b-D-thiogalactopyranoside at 18 °C. His-Tagged BRD4 ET and His-Tagged JMJD6 were purified by HiTrap IMAC (GE Healthcare). After removing His-Tag with thrombin or TEV treatment, protein samples were further purified with Superdex 75 column or Superdex 200 column (GE Healthcare). Uniformly ^15^N-, ^15^N/^13^C-labeled proteins were prepared by growing the bacteria in M9 minimal medium containing ^15^NH_4_Cl with or without ^13^C-glucose and prepared as unlabeled protein.

### Nuclear Magnetic Resonance Spectroscopy

The BRD4 ET domain/JMJD6 peptide (84–96) complex was used for structure determination. NMR samples of the ET domain (0.5 mM) in complex with a JMJD6 peptide (Residues 84–96, 13-mer, KWTLERLKRKYRN) of 1.0 mM were prepared in 100 mM phosphate buffer (pH 6.5) containing 5 mM perdeuterated DTT and 0.5 mM EDTA in H2O/^2^H_2_O (9/1) or ^2^H_2_O. All nuclear magnetic resonance (NMR) spectra were acquired at 25 °C on Bruker 500, 600, and 800 MHz spectrometers equipped with z-gradient triple-resonance cryoprobes. The backbone ^1^H, ^13^C, and ^15^N resonances were assigned using standard three-dimensional triple-resonance HNCA, HN(CO)CA, HN(CA)CB, and HN(COCA)CB experiments^34^. The side-chain atoms were assigned from three-dimensional HCCH-TOCSY, HCCH-COSY, and (H)C(CO)NH-TOCSY data^35^. The NOE derived distance restraints were obtained from ^15^N- or ^13^C-edited three-dimensional NOESY spectra. The JMJD6 peptide was assigned from two-dimensional TOCSY, NOESY, ROESY, and ^13^C/^15^N-filtered TOCSY and NOESY. The intermolecular NOEs used in defining the structure of the complex were detected in ^13^C-edited (F1), ^13^C/^15^N-filtered (F3) three-dimensional NOESY spectra (unlabeled JMJD6 peptide bound to ^13^C/^15^N-labeled ET protein)^36^. Spectra were processed with NMRPipe and analyzed using NMRVIEW^37,38^.

### Structure Calculations

Structures of the BRD4 ET domain/JMJD6 peptide were calculated with a distance-geometry simulated annealing protocol with CNS^39^. Initial protein structure calculations were performed with manually assigned NOE-derived distance constraints. Hydrogen bond distance, ϕ and ψ dihedral-angle restraints from the TALOS-N prediction were added at later stage of structure calculations for residues with characteristic NOE patterns^40,41^. The converged structures were used for the iterative automated NOE assignment by ARIA refinement. Structure quality was assessed with CNS, ARIA, and PROCHECK analysis^42,43^. Total 143 intermolecular NOE-derived distance restraints were added in the structure determination of the ET/JMJD6 peptide complex. A family of 200 structures was generated and 20 structures with the lowest energies were selected for the final analysis.

### Isothermal Titration Calorimetry

Experiments were carried out on a MicroCal auto-ITC200 instrument at 20 °C while stirring at 750 rpm in ITC buffer (pH 7.4), consisting of PBS buffer and 0.5 mM dithiothreitol as described previously^27,44^. Peptide concentration was determined by weight and confirmed by NMR, and protein concentration by absorbance measured at 280 nm (A280). The protein sample (0.5 mM) was placed in the cell, whereas the micro-syringe was loaded with a peptide (7.5 mM) in the ITC buffer. The titrations were conducted using 19 successive injections of 2.0 μl (the first at 0.4 μl and the remaining 18 at 2.4 μl) with a duration of 4 s per injection and 150 s between injections. The collected data were processed using the Origin 7.0 software program (OriginLab) supplied with the instrument according to the “one set of sites” fitting model.

### Differential Scanning Fluorimetry (DSF) – Thermal Shift Measurements

DSF measurements were performed using a method described by^45^. SYPRO Orange dye (Life Technologies) was incubated at ratio of 1:1000 (w/w) with ~0.05 mg/mL of full-length JMJD6 (1 μM) in PBS (pH 7.4). If samples also include the ET domain, the concentrations were 1 μM to 100 μM. If samples also include ssRNA, dsRNA, ssDNA, or dsDNA, those concentrations were 1 μM, respectively. All oligonucleotides were purchased from eurofins. The sequence of ssRNA was 5′-AUACGAUGCUUUACGGUGCUAUUUUGU-3′; 27 nt. The sequence of ssDNA was 5′-TTTCTAGATCAGGAGCTGTCGGAAGCTATCC-3′; 31 nt. 40 μL of the dye/protein solution was aliquoted into a 96-well PCR plate, and an emission of 610 nm was measured in a real-time PCR instrument (Strategene Mx3005P, Agilent Technologies, Waltham, MA, USA) as the temperature was increased from 25 °C to 95 °C at 1 °C/min. The fluorescence was fit by regression analysis using IGOR Pro (WaveMetrics, Inc., Lake Oswego, Oregon, USA) using a simple equation such as the Boltzmann equation,

1 $$y=LL+(UL-LL)/\{1+\exp ({T}_{m}-x)/a\},$$

where LL and UL are lower and upper baselines with the slope and intercept, respectively, and *a* denotes the slope of the curve within the inflection point of the transition curve (*T*_m_). Using *T*_m_ and *a* obtained by the fitting, the thermal shift data were normalized to remove temperature dependency of fluorescent intensities and effect of protein aggregation that distort an intrinsic transition curve.

### Plasmid Constructions

The mammalian expression plasmid, pcDNA3-F:hBrd4 (FL) and pcDNA3-F:hBrd4 (E651A E653A) were gifts from MMZ lab. To generate ET domain deletion mutant of hBrd4, we used pcDNA3-F:hBrd4 (FL) as template. There are two unique restriction enzyme cleavage sites near ET domain, Bsu36I and AleI. Overlap extension PCR was employed to generate domain-specific deletion of ET by using a pair of primers flanking the region where the deletion will be made (primers 1 and 4), and two complementary primers comprising a region of −19 bp to +26 bp related to the junction point (primers 2 and 3). The amplified product was then digested with Bsu36I and AleI at 37 °C for 3 h for subsequent cloning. The resulting clone, after bacterial transformation and DNA sequencing, was named pcDNA3-F:hBrd4 (ET-deletion).

### Co-immunoprecipitation

The day before performance of transfections, cells were seeded such that they would reach 70% confluence at the time of transfection. HEK-293T cells were transfected with the indicated plasmids using a 3:1 PEI/DNA ratio according to the instructions of the manufacturer (Polysciences, CAT#23966-2). After 48 hours transfection, cells were lysed in lysis buffer (20 mM Tris-HCl pH 7.5; 150 mM NaCl; 0.5% NP-40; 2mM MgCl2) supplemented with protease inhibitor cocktail (Roche). Cell lysates were sonicated at 30% intensity for 6s (3s on and 3s off and repeat twice) and clarified by spinning at 14000rpm for 15 min. Add same volume of IP binding buffer (20 mM Tris-HCl pH 7.5; 150 mM NaCl; 0.5 mM EDTA) to lysate, then add anti-Flag (Flag M2 resin; Sigma A2220) or anti-Brd4 (Bethyl A301-985A) and pre-cleared protein G dynabeads (Thermo Fisher). Incubate at 4 °C overnight. After 5 washes with the wash buffer (20 mM Tris-HCl pH 7.5; 150 mM NaCl; 0.5 mM EDTA, 0.02% tween-20), the bound protein was eluted in SDS Laemmli buffer and size fractionated on Bis-Tris gels prior to blotting with the indicated antibodies. For immunoprecipitation adding Single-stranded RNA(ssRNA), we also need to add ssRNA and RNase inhibitor (RNasin, Promega) to the cell lysate.

### Accession Numbers

PDB: coordinates for the solution structures of the BRD4 ET domain in complex with JMJD6 peptide is deposited at PDB under accession numbers PDB: 6BNH, and the NMR spectral data are deposited at BioMagResBank (BMRB) under accession numbers BMRB: 30373, respectively.

## Electronic supplementary material

Supplementary Information
