# Supplementary material for: Structural Mechanism of the Oxygenase JMJD6 Recognition by the Extraterminal (ET) Domain of BRD4
Source: Sci Rep. 2017 Nov 24;7:16272. doi: 10.1038/s41598-017-16588-8 (PMC5701133; doi:10.1038/s41598-017-16588-8)
Supplement: Supplementary file 1 — Supplementary Information [file 41598_2017_16588_MOESM1_ESM.pdf]

# Structural Mechanism of the Oxygenase JMJD6 Recognition by the Extraterminal (ET) Domain of BRD4

Tsuyoshi Konuma*[1], Di Yu*[2], Chengcheng Zhao[2], Ying Ju[2], Rajal Sharma[1], Chunyan Ren[1], Qiang Zhang[2,1], Ming-Ming Zhou[1] & Lei Zeng[2,1]

[1]Department of Pharmacological Sciences, Icahn School of Medicine at Mount Sinai, New York, NY, 10029, USA
[2]Bethune Institute of Epigenetic Medicine, The First Hospital, Jilin University, Changchun, Jilin, 130021, China

*: Both authors contributed equally to this work.

Correspondence: lei.zeng02@qq.com

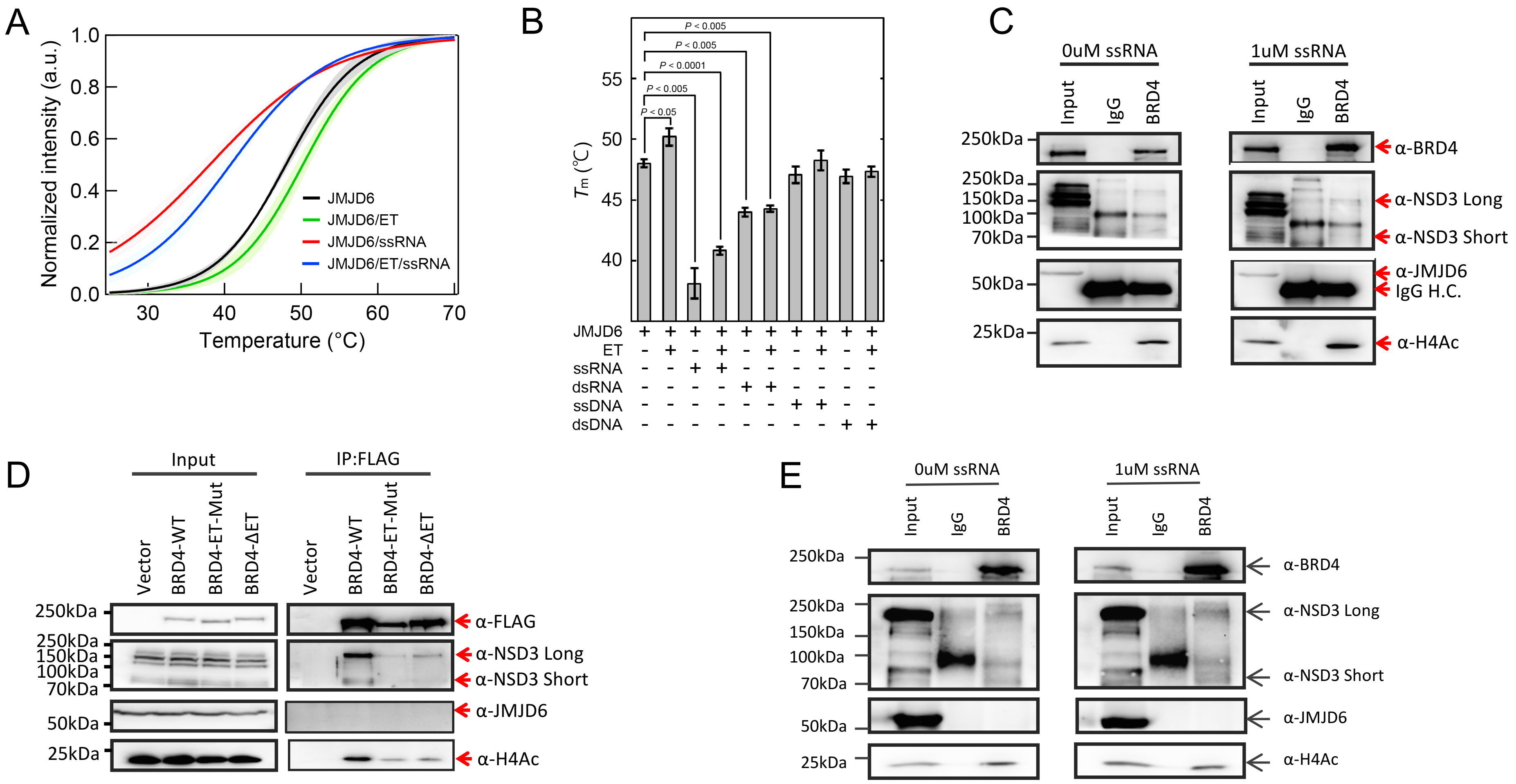


Supplemental Figure 1, (A) Thermal stability of the full-length JMJD6 determined by differential scanning fluorimetry with concentrations of JMJD6 at 1μM and ET at 1μM. The averaged fluorescent intensities were normalized using $T_m$ and $a$ obtained by fitting thermal shift data to a theoretical equation (see Experimental procedures). The profiles in black, green, red and blue were obtained from free JMJD6, JMJD6/ET, JMJD6/ssRNA and JMJD6/ET/ssRNA, respectively. The standard deviations of the profiles were calculated from three independent experiments. In control experiments, samples of only ET, ssRNA, dsRNA, ssDNA or dsDNA without JMJD6 had no fluorescent intensity; (B) Thermal stability chart of JMJD6 with/without ET, ssRNA, dsRNA, ssDNA and dsDNA. $T_m$ is presented as mean ± SEM (n = 3). P-values calculated by the t-test indicate a statistically significant difference; (C) Endogenous IP experiments. Nuclear extracts added with or without ssRNA purified from HEK293T cells were subjected to immunoprecipitation (IP) and followed by western blotting; (D) HEK293T cells were transfected with Flag-BRD4WT, Flag-BRD4-Mut and Flag-BRD4-ΔET and cell lysates were treated with ssRNA and subjected to IB analysis; (E) HEK293T cells were transfected with His-JMJD6 and subjected ssRNA treatment and IB analysis.

# Supplemental Figure 2, ITC binding affinity assay of JMJD6 peptides

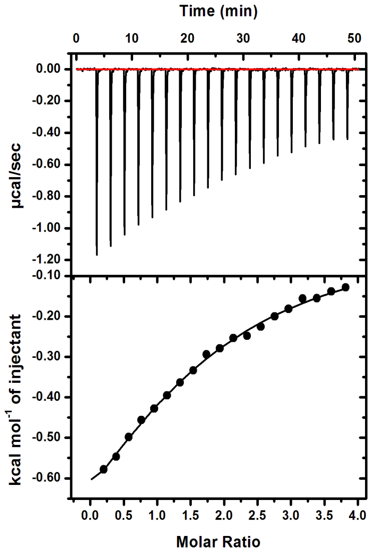


W85A

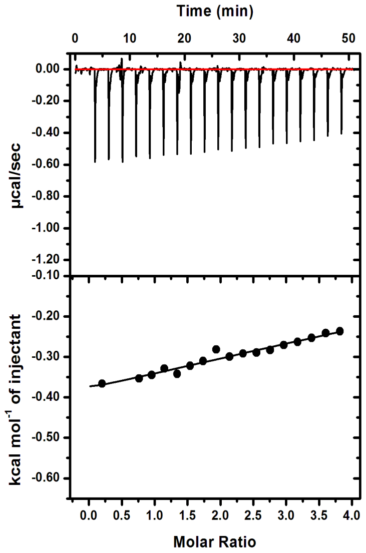


L90A

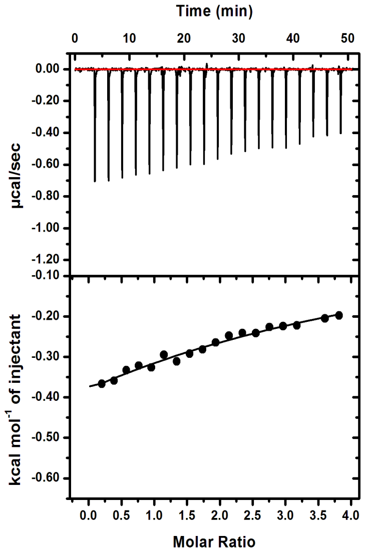


K91A

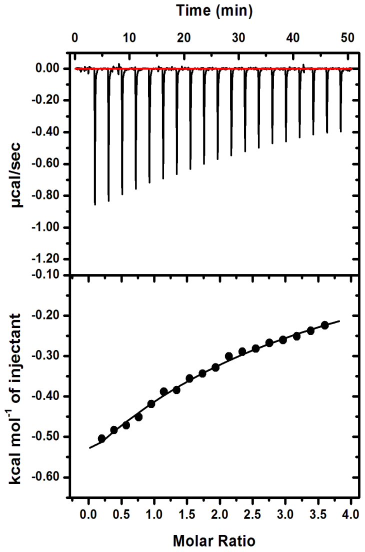


R95A

| Peptide Name | Peptide Sequence | ITC Binding (Kd) |
|---|---|---|
| JMJD6 WT | KWTLERLKRKYRN | 158 ± 14 µM |
| JMJD6 W85A | K**A**TLERLKRKYRN | 675 ± 66 µM |
| JMJD6 L90A | KWTLER**A**KRKYRN | No Binding |
| JMJD6 K91A | KWTLERL**A**RKYRN | No Binding |
| JMJD6 Y94A | KWTLERLKRK**A**RN | N/A |
| JMJD6 R95A | KWTLERLKRKY**A**N | No Binding |

Supplemental Figure 3, $^{15}$N-HSQC titration of JMJD6 peptide with ET domain in the PBS buffer of pH7.5 at 298K, black color spectrum is free protein, red is JMJD6/ET complex. The protein concentration is 0.1mM and the molar ratio of the protein to peptide is 1:5.

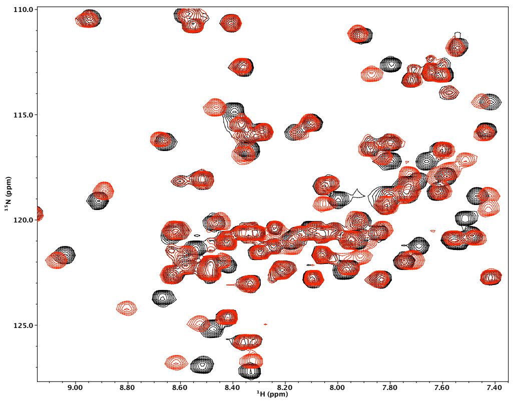


Wild type

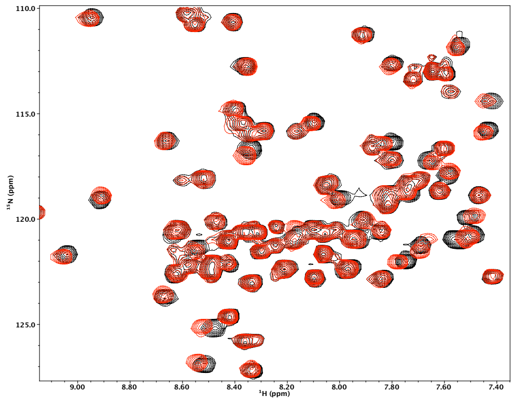


W85A

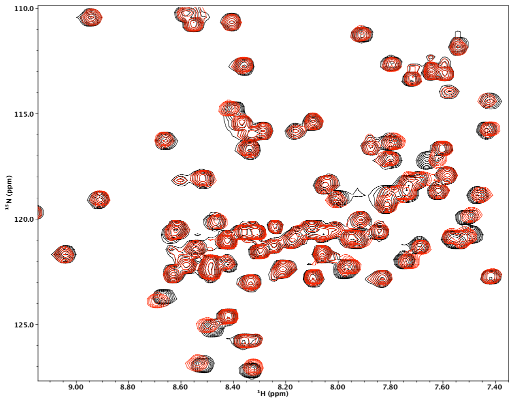


L90A

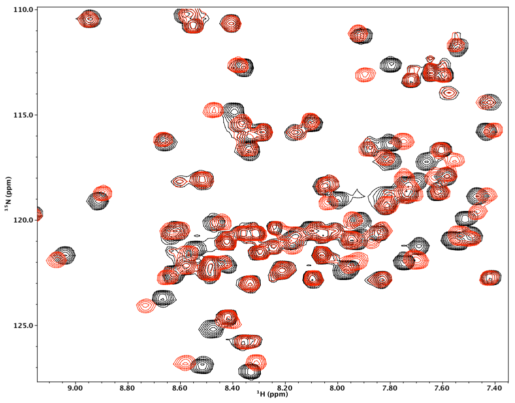


K91A

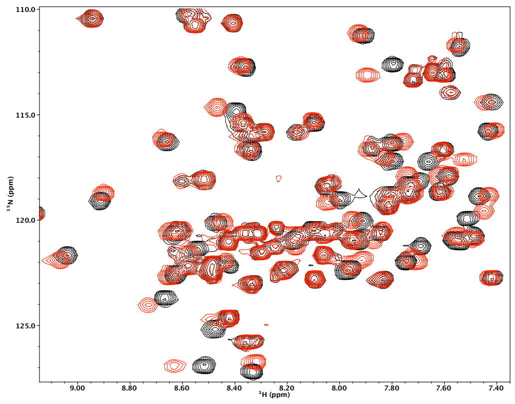


R95A

| **Peptide Name** | **HSQC titration** |
|---|---|
| JMJD6 WT | Good binding |
| JMJD6 W85A | Very weak |
| JMJD6 L90A | No Binding |
| JMJD6 K91A | weak |
| JMJD6 Y94A | N/A |
| JMJD6 R95A | weak |
